# A Light Field Full-Focus Image Feature Point Matching Method with an Improved ORB Algorithm

**DOI:** 10.3390/s23010123

**Published:** 2022-12-23

**Authors:** Ying Zuo, Hongliang Guan, Fuzhou Duan, Tingsong Wu

**Affiliations:** 1 Engineering Research Center of Spatial Information Technology, Ministry of Education, Capital Normal University, 105 West Third Ring North Road, Beijing 100048, China; 2 School of Resource and Environment Sciences (SRES), Wuhan University, 129 Luoyu Road, Wuhan 430079, China; 3 Key Lab of 3D Information Acquisition and Application, Ministry of Education, Capital Normal University, 105 West Third Ring North Road, Beijing 100048, China; 4 Beijing Imaging Technology Innovation Center, Capital Normal University, 105 West Third Ring North Road, Beijing 100048, China

**Keywords:** light field, full-focus image, feature point extraction, threshold adaptive, feature point matching

## Abstract

Most of the traditional image feature point extraction and matching methods are based on a series of light properties of images. These light properties easily conflict with the distinguishability of the image features. The traditional light imaging methods focus only on a fixed depth of the target scene, and subjects at other depths are often easily blurred. This makes the traditional image feature point extraction and matching methods suffer from a low accuracy and a poor robustness. Therefore, in this paper, a light field camera is used as a sensor to acquire image data and to generate a full-focus image with the help of the rich depth information inherent in the original image of the light field. The traditional ORB feature point extraction and matching algorithm is enhanced with the goal of improving the number and accuracy of the feature point extraction for the light field full-focus images. The results show that the improved ORB algorithm extracts not only most of the features in the target scene but also covers the edge part of the image to a greater extent and produces extracted feature points which are evenly distributed for the light field full-focus image. Moreover, the extracted feature points are not repeated in a large number in a certain part of the image, eliminating the aggregation phenomenon that exists in traditional ORB algorithms.

## 1. Introduction

Image feature point extraction and matching is an important task in computer vision technology and is widely used in the fields of 3D reconstruction, visual SLAM, and face recognition. An accurate feature point extraction and matching results are crucial for the development of computer vision technology. Since the 1990s, intensive research on image feature point extraction and matching has largely advanced the development of computer vision technology. At the same time, the frontier fields of computer vision, such as target detection, 3D reconstruction, full-scene stitching, and SLAM, have generated tougher requirements on the accuracy and robustness of image feature point extraction and the matching methods. Researchers have conducted numerous studies on the traditional image feature point extraction and matching methods and have also achieved numerous results. These include Harris [1], SIFT [2], HOG [3], SURF [4], FAST [5], BRIEF [6], and ORB [7]. These methods are basically based on a series of the light properties of the image. This light property easily conflicts with the distinguishability of the image’s features, which leads to poor feature matching results. The reason for this is that the traditional light imaging methods can record only information about the position of light at one angle in the target scene, which can easily obscure information about the target at other locations. The traditional light imaging method focuses only on a fixed depth of the target scene, and subjects at other depths are often easily blurred. Moreover, the traditional image feature point extraction and matching methods have great challenges in dealing with scenes that contain shadows, similar textures, and luminance variations, for example. The existence of these problems greatly limits the development of traditional image feature point extraction and matching algorithm research.

Light field imaging is a technology that performs imaging first and then uses algorithms to calculate the imaging again [8,9]. It integrates the traditional light imaging, signal processing, and depth extraction methods [10]. The light field acquisition device can capture the 4D information of light and store it while acquiring subaperture images from multiple angles with a single exposure. It has good prospects for applications in depth extraction, 3D modeling, and virtual reality. The traditional light camera can record only light information at one angle, while the light field camera can record both the position information and direction information of light in the target scene, which makes it easy to extract the depth information of the scene. The light field camera has the characteristic of “taking pictures first, focusing later”. This allows the subsequent refocusing algorithm to acquire images at any depth position and then obtain a light field full-focus image. Then, the extraction and matching of the image’s feature points are realized by using the high resolution of the light field full-focus image. This approach is expected to address the problems in the current research of image feature point extraction and matching methods.

In this paper, a light field camera is used as a sensor to acquire image data. Then, the full-focus image is generated with the rich depth information inherent in the original light field image. Finally, the extraction and matching of the image’s feature points are achieved using the light field full-focus image. The main contributions of this paper are as follows.

(1) The high-quality depth information in the target scene is totally acquired by fusing multiple depth cues. Then, by using this depth information as an index, the corresponding pixel values are collocated to obtain the light field full-focus image.
(2) The feature points are extracted and matched with the full-focus image generated by the light field depth information, and these feature points have not only a general scale invariance and rotation invariance but also depth characteristics.
(3) A threshold adaptive method is proposed to improve the accuracy of the feature point extraction, and a large number of duplicate feature points are eliminated by using nonmaximal value suppression.

## 2. Related work

### 2.1. 2D Image Feature Point Extraction and Matching

Researchers have conducted much research on the feature point extraction and matching methods for 2D images. Moravec proposed the earliest image feature point extraction algorithm, the Moravec corner detector, based on the process of mobile robot motion state estimation [11]. On this basis, Matthies implemented the extraction and tracking of the feature points by using a binocular vision system combined with the Moravec corner detector algorithm [12,13]. The image feature point extraction and matching methods from this period were mainly based on the corner point detection algorithms. Typical corner point detection operators also include the Forstner [14] and SUSAN [15] operators. To extract feature points that are not affected by image rotation and scaling, Lowe proposed the SIFT feature extraction algorithm. The algorithm is able to select the true feature points from the set of other candidate feature points [2]. However, the above feature extraction algorithms have problems such as a high computational complexity and a long computation time. For this, Rosten proposed the FAST feature extraction algorithm. The best advantage of this algorithm is its very high computational efficiency and its ability to perform processing in real time [5]. The FAST feature extraction algorithm greatly improves the efficiency of the image feature point extraction but is poor in terms of its accuracy. To balance the efficiency and accuracy of the image feature extraction, Rublee proposed the ORB feature extraction algorithm by combining the FAST and BRIEF algorithms. The ORB algorithm adds a combination of rotational invariances to the combination of the two algorithms while achieving a scale invariance with scale pyramids [7].

### 2.2. Light Field Image Feature Point Extraction and Matching

Light field cameras are able to record both the direction and intensity of light compared to the ability of traditional cameras. This allows us to obtain some images of virtual perspectives through computational imaging. Based on this, researchers have started to explore the feasibility of applying light field imaging technology to image feature point extraction and matching methods. Tosic proposed a method to detect the 3D feature points from light field data based on the constructed depth space of the light field scale [16]. However, this method does not work well for scenes with occlusion. Texeira proposed a feature detector for microlens light field images based on para-polar planar images (EPIs). The detector uses the SIFT algorithm for the feature detection of light field subaperture images and then maps the detected candidate feature points into the corresponding EPI. Subsequently, the detector performs a line segment detection in the EPI, thereby determining the final feature points, which improves the accuracy of the feature detection [17]. To address the challenge of a feature detection and description due to the light transmission effect, Dansereau proposed LIFF 4D light field feature extraction and descriptors that use the spatial angle imaging mode of light field cameras. This descriptor has a scale invariance and is highly robust for detecting features with perspective changes [18]. To improve the accuracy of the feature extraction, Assaad proposed a method to detect the feature points on the original light field image by applying a feature point detector on the pseudofocused image (PFI) [19]. However, the above methods require a high computational cost and greatly increase the time complexity of the algorithm. Therefore, although the feature point extraction and matching methods for 4D light field images have improved the accuracy and precision of the image feature point extraction and matching methods to a certain extent, the problems of a low efficiency and a high time complexity of the feature point extraction and matching algorithms due to the large amount of data in 4D light field images still exist.

## 3. Methods

### 3.1. Light Field Full-Focus Image Generation Method Fusing Multiple Cues

The amount of original 4D light field image data captured with the light field camera is relatively large due to its special structure and sensor, which leads to the long computation time required for the light field image feature point extraction and matching. Therefore, this paper proposes a light field image processing method to integrate the light field data, which not only can obtain a higher clarity light field full-focus image but also can greatly reduce the volume of the data. Thus, the time for a light field image feature point extraction and matching is reduced with a guaranteed accuracy.

The light field camera has the characteristic of “take a picture first, focus later”. That is, the shutter can acquire a series of images with different focal points in one exposure. In this paper, we use this series of images to generate a focus stack image and obtain high-quality depth information of the target scene from the focus stack image. Then, the high-quality depth information is used to obtain the focus area in the focus stack image, and the light field full-focus image is obtained from this area. The light field focus stack is actually a collection of multiple images focused at different target scene depths [20]. Notably, the light field focus stack is not simply a series of refocused images. These refocused images are identical in all parameters except for the depth information of the images. A series of images in a light field focus stack each has its own depth of field. The collection of these depths of field together makes up the depth of the field of the light field focus stack. This allows the light field focus stack to contain rich image information, including image depth information and spatial information. Thus, it is possible to obtain images with a greater depth of field from the light field focus stack by fusion, which can be used as the basis for extracting light field depth information to generate light field full-focus images.

**Figure 1:**
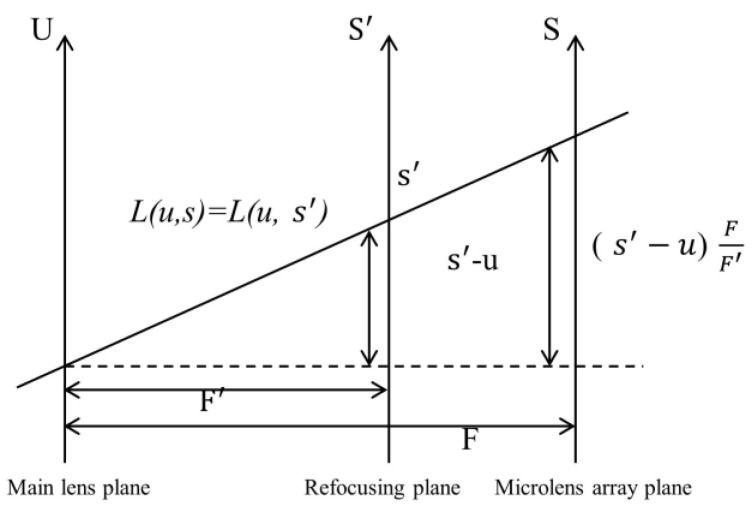
Refocusing principle schematic.

The light field focus stack is built on the basis of light field refocusing. The original 4D light field image is digitally refocused and then rendered by integration to obtain images focused at different depths. The collection of images focused at different depths is the focus stack image. The light field refocusing model used in this paper produces the relationship between the pixel points after changing the focus plane by the proportional relationship of similar triangles. The principle of digital refocusing in this way is to calculate the intensity of the obtained light field information on a new imaging plane. This new imaging plane is calculated from the coordinates corresponding to the main lens plane and the microlens array plane in the vertical direction. The schematic diagram is shown in Figure 1.

The refocusing principle can be expressed as follows.

(1) $$E\left(s^{\prime },t^{\prime },alpha\right)=\frac{1}{\alpha^{2}F^{2}}\iint L_{s}\left[u,v,u\left(1-\frac{1}{\alpha }\right)+\frac{u^{\prime }}{\alpha },v\left(1-\frac{1}{\alpha }\right)+\frac{v^{\prime }}{\alpha }\right]dudv$$

where *F* is the distance between the main lens plane and the microlens array plane and $F^{\prime }$ is the distance between the main lens plane and the refocusing plane. Each refocused image has a fixed α value, so the light field can be expressed as $L\left(x,y,dr,dz\right)$. x×y represents the spatial resolution of the light field, and each dr value corresponds to an α value, representing a depth. $dz=\left(\frac{u_{max}}{2},\frac{v_{max}}{2}\right)$ indicates that it is the central subaperture field of view, and $L\left(x,y,dr,dz\right)$ can be reduced to $L{\left\{x,y,dz\right\}}_{dr=1}^{max}$ without considering the field of view.

The depth information of the object refers to the distance between the object and the main lens. Commonly used image depth recovery methods include the gradient cue recovery method, the scattered-focus cue recovery method, and the parallax cue recovery method. The gradient method has difficulty obtaining a depth cue with a good effect in the global range because the images are close to each other, causing the gradient value of the images to vary less significantly. Although the scattered-focus method shows a good stability in target scenes with repeated textures, the contrast changes in the image edge regions due to changes in the brightness can easily lead to errors in the results, making the depth cues in these regions unable to be accurately obtained. The parallax method is able to reduce the error of the scattered-focus method, but due to the limitation of the search space, matching ambiguity ensues when the image movement is large. In particular, more matching ambiguities occur in scenes with occlusion and noise, which leads to less accurate depth cues in these scenes. Therefore, there are many problems in using a single method to obtain the depth information of light field images. Fusing multiple depth cues to obtain the depth information is expected to yield a result with a high accuracy, good quality, and robustness. The 4D light field image contains rich information about the depth of the light field, and the degree of blurring in the unfocused region of the refocused image in the focus stack image contains very rich clues about the depth of the scattered focus. The multiple subaperture images of the light field images have many redundancies of the image depth cues, which implies a very rich parallax depth cue. Based on this, in this paper, we break through the limitation of using only a single recovery method in the traditional depth information acquisition method and fuse multiple depth cue recovery methods to obtain high-quality light field depth information. This depth information is used as an index to obtain the focus area in the focus stack image, to calculate the values of all the pixel points in the focus area, and then to recombine these pixel values to obtain the light field full-focus image.

The scattered-focus cue for all the depth images in the focus stack is $D\left(x,y,\alpha \right)$, and the parallax cue is $C\left(x,y,\alpha \right)$. The calculation results of these two cues may have different representations. To minimize the fusion error, before fusing the two depth cues, a maximum–minimum normalization process is needed to preprocess the computed results of the two depth cues, that is, to traverse the computed results of the two depth cues pixel-by-pixel. Once the results are normalized, it is necessary to fuse the two depth cues of the scattered focus and parallax. To minimize the errors due to the parameters and other reasons, we use the confidence level to fuse the computed results of the two depth cues. The confidence level of both depth cues can be calculated using the peak ratio method [21]. For any pixel point in space, the scattered-focus and parallax cues of that point in the light field focus stack image are first calculated, and the maximum and second maximum values of the scattered-focus cues and the minimum and second minimum values of the parallax cues are calculated. The formula for calculating the confidence level of the two depth cues is shown below.

(2) $$C_{D}\left(x,y\right)=\frac{D_{\alpha_{1}}\left(x,y\right)}{D_{\alpha_{2}}\left(x,y\right)}$$

(3) $$C_{C}\left(x,y\right)=\frac{C_{\alpha_{1}}\left(x,y\right)}{C_{\alpha_{2}}\left(x,y\right)}$$

where $D_{\alpha_{1}}\left(x,y\right)$ is the maximum value of the scattered-focus response, $D_{\alpha_{2}}\left(x,y\right)$ is the second maximum value, $C_{\alpha_{1}}\left(x,y\right)$ is the minimum value of the parallax response, and $C_{\alpha_{2}}\left(x,y\right)$ is the second minimum value.

The confidence level used in this paper is the weight when fusing the scattered-focus and parallax depth cues, and the higher the value of the confidence level is, the higher the confidence level of that depth cue. The calculation formula is shown below.

(4) $$C\left(x,y,\alpha \right)=C_{C}{\left(x,y\right)}^{\lambda_{C}}\times C^{\prime }\left(x,y,\alpha \right)+C_{D}{\left(x,y\right)}^{\lambda_{D}}\times \left[1-D^{\prime }\left(x,y,\alpha \right)\right]$$

In the formula, $\lambda_{C}$ is the coefficient of the contribution of the scattered-focus response to the depth estimate, which is taken as 0.2; $\lambda_{D}$ is the coefficient of the contribution of the parallax response to the depth estimate, which is taken as 0.6.

The depth parameter used in this paper is the minimum value after a pixel-by-pixel search for multicue fusion. The calculation formula is shown below.

(5) $$d=\arg\underset{\alpha }{\min}C\left(x,y,\alpha \right)$$

**Figure 2:**
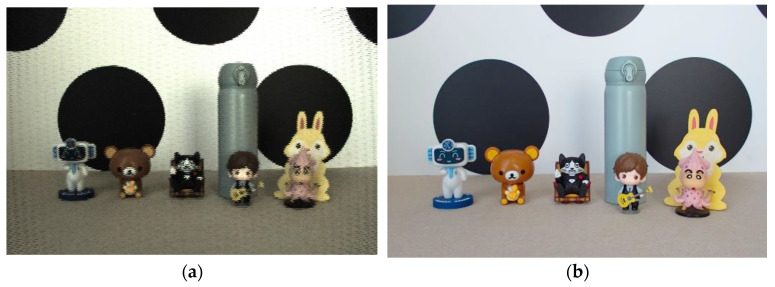
Scene 1 light field full-focus image. (**a**) The original images of the light field. (**b**) The full-focus images of the light field.

**Figure 3:**
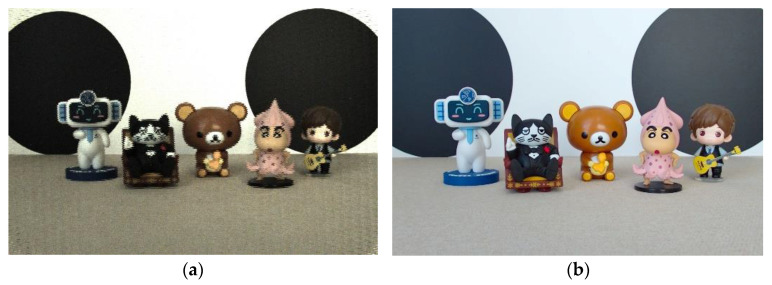
Scene 2 light field full-focus image. (**a**) The original images of the light field. (**b**) The full-focus images of the light field.

**Figure 4:**
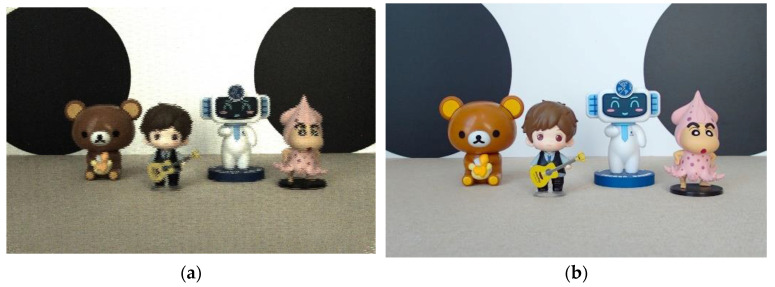
Scene 3 light field full-focus image. (**a**) The original images of the light field. (**b**) The full-focus images of the light field.

**Figure 5:**
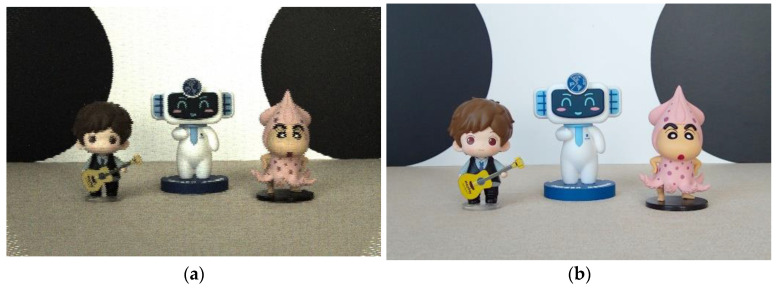
Scene 4 light field full-focus image. (**a**) The original images of the light field. (**b**) The full-focus images of the light field.

We suppose the final depth map obtained by using the two-cue fusion method is *d*(*x*, *y*). For each pixel (*x*, *y*), the depth map is treated as a depth index map, and according to the depth index map, the corresponding focus region is determined from the focus stack. Then, the full-focus image $f\left(x,y\right)$ is generated by extracting and integrating from the focus region. The specific formula is shown below. The final full-focus images generated in this paper for the four experimental scenes are shown in Figure 2, Figure 3, Figure 4 and Figure 5. In the following figures, the left images are the original images of the light field, and the right images are the full-focus images of the light field.

(6) $$f\left(x,y\right)=L\left(x,y,d_{r}\right)=L\left(x,y,d\left(x,y\right)\right)$$

Clarity is an important indicator to evaluate the image quality. The light field full-focus image acquired by fusing the two depth cues in this paper has a high clarity from the visual point of view. To show the effect of the image more intuitively, we use the image clarity index to quantitatively evaluate the image quality. To better show the effect of the experiment, the experimental results of the light field central subaperture images were added as a control group.

The gradient of the image edge can often reflect the degree of sharpening and clarity of the image. Therefore, most of the commonly used image clarity evaluation functions use the gradient function to calculate the image edge information and use the gradient function value to evaluate the image clarity. The commonly used gradient functions include the Tenengrad function, the Laplace function, and the variance function, among which the most commonly used is the Tenengrad function; thus, we use the Tenengrad function to evaluate the clarity of the acquired light field full-focus images. The Tenengrad function uses the Sobel operator to calculate the gradient values of a pixel point in the horizontal and vertical directions and considers the sum of the squares of the two gradient values as the clarity of the point, and the gradient values of each pixel point can be accumulated to obtain the clarity of the whole image [22]. The process is as follows: we suppose the convolution kernel of the Sobel operator is $G_{X}$, $G_{y}\;$; then, the gradient of image *I* at point (*x*, *y*) can be expressed as Equation (7).

(7) $$S\left(x,y\right)=\sqrt{G_{x}\times I\left(x,y\right)+G_{y}\times I\left(x,y\right)}$$

Then, the Tenengrad value of the image is calculated as shown in Equation (8).

(8) $$Ten=\frac{1}{n}\underset{x}{\sum }\underset{y}{\sum }S{\left(x,y\right)}^{2}$$

In the formula, *n* is the total number of pixels in the image.

Finally, the Tenengrad values of the light field full-focus image and the light field central subaperture image calculated in this paper are shown in Table 1.

As shown in the table above, the Tenengrad values of the light field full-focus images acquired in this paper are higher than the Tenengrad values of the light field central subaperture images for the four experimental scenes involved. The light field full-focus images acquired in this paper are not only clearer but also have sharper edges.

### 3.2. Image Feature Point Extraction and Matching Method with Elimination of Aggregation

The ORB image feature extraction and matching algorithm is widely used in computer vision technology because of its fast extraction speed and guaranteed extraction accuracy. However, the threshold used by the traditional ORB algorithm in the process of extracting the feature points is a fixed value set artificially, which means that all images that have their feature points extracted have the same value of the threshold set in the process of extracting the feature points, which is obviously unreasonable. Since the parameters of all the images are not identical, inconsistencies in some of the parameters of some images lead to large errors in the feature extraction and matching results. Based on this, we propose a threshold adaptive method to set the threshold according to the gray value of the surrounding range of each point to be extracted so that the stability of the algorithm can be guaranteed in the face of changes in the gray value of the image. The method is specifically calculated by setting the coordinates of the feature point P on the image to be extracted as $\left(x_{0},y_{0}\right)$ and by defining the threshold T with the variation in the gray values in the image. This is shown in Equation (7).

(9) $$T=k\times \frac{\sum_{i=1}^{n}\left(I_{i_{max}}-I_{i_{min}}\right)}{\sum_{i=1}^{n}\left(I_{i_{max}}+I_{i_{min}}\right)}$$

In the formula, $I_{i_{max}}$ denotes the maximum *n* gray values in the square region, $I_{i_{min}}$ denotes the minimum *n* gray values in the square region, and *k* is the scale factor; here, *k* = 20 is used.

After the feature points are filtered, the filtered feature points tend to be aggregated because there are many pixel points near the real feature point that are very similar to the point, and the FAST algorithm sometimes cannot accurately distinguish between them. For this, the nonmaximum suppression method is used in this paper to further filter the true feature points. The specific steps of the method are as follows.

(1) When the FAST feature extraction algorithm identifies a pixel point (*x*, *y*) as a candidate feature point, it calculates the absolute value of the difference between the gray value of that point and the 16 pixel points on its corresponding circle.
(2) We sum the results for 16 absolute values.
(3) The score function is used to determine the scores for all the candidate feature points (*x*, *y*). The specific process is shown in Equation (8).

(10) $$score\left(x,y\right)=\overset{16}{\underset{i=1}{\sum }}\left|I\left(x,y\right)-I_{i}\right|$$

After the nonmaximal suppression method is used to calculate the scores of all the candidate features, the scores of the two neighboring candidates are compared. The pixel with the smaller score is removed from the set of candidate feature points, and the remaining candidate feature points are the score maxima in the local area. In this way, the problem of a feature point aggregation in the region is solved.

The traditional ORB algorithm leads to a large number of mis-matches due to the presence of some repeated texture features in the image; the modifications made in this paper based on this are as follows.

(1) The descriptors of the feature points are computed by the image after Gaussian blurring.
(2) For the matched feature points, the relative rotation angle is calculated using their orientations derived from the grayscale center of mass, and then the matches with different rotation directions from the mainstream are rejected to weed out the mis-matches.
(3) In the process of searching for a match, the Hamming distance of the searched pair of optimal matches must not only be less than a set threshold but must also be significantly smaller than the Hamming distance of the suboptimal matches. Ultimately, the probability of feature point mis-matches is reduced.

## 4. Experiments

**Figure 6:**
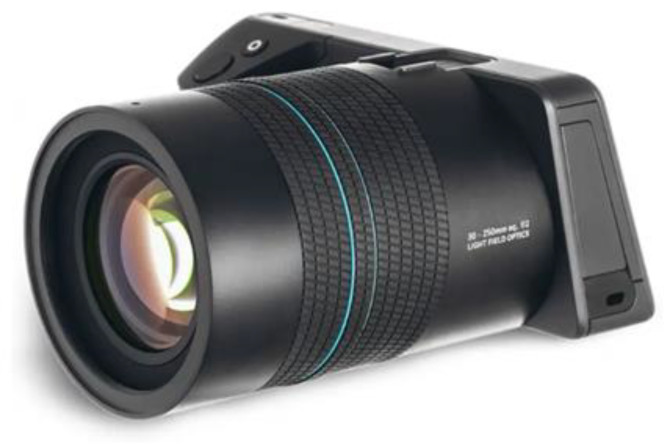
Lytro Illum light field camera.

The light field images used in this paper were captured by the light field camera Lytro Illum, as shown in Figure 6. The light field camera Lytro Illum produces approximately 40 million effective pixels, the capture sensor is 7728 × 5368, the microlens array is 541 × 434, the angular resolution is 15 × 15, and the number of pixels behind each microlens is 225.

**Figure 7:**
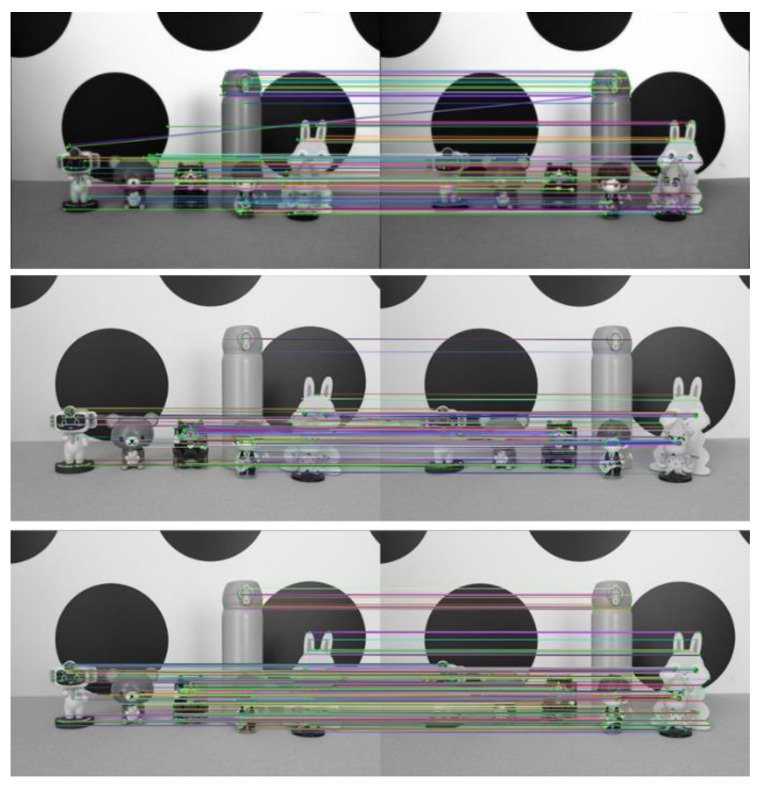
Scene 1 image feature point matching results.

To verify the advantages of the light field full-focus image acquired in this paper over the traditional image in terms of the feature point extraction and matching, we use the light field central subaperture image to perform the feature extraction and matching at the same time. Then, the improved ORB algorithm is used to extract and match the features of the light field full-focus image obtained in this paper. The advantages of the improved algorithm over the traditional ORB algorithm are compared and analyzed. The experimental results of the three parts are shown in Figure 7. In the figures, the first figure shows the experimental results of the light field central subaperture image feature point matching using the traditional ORB algorithm, the second figure shows the experimental results of the light field full-focus image feature point matching using the traditional ORB algorithm, and the third figure shows the experimental results of the light field full-focus image feature point matching using the improved ORB algorithm.

The number of feature point matching pairs achieved on the light field central subaperture image by using the traditional ORB algorithm is 112. The number of feature point matching pairs achieved on the light field full-focus image by using the traditional ORB algorithm is 190. The number of feature point matching pairs achieved on the light field full-focus image by using the improved ORB algorithm is 739. With the same method, more matching pairs are achieved using the light field full-focus image than using the light field central subaperture image. For the same image, more matching pairs are also achieved using the improved ORB algorithm than using the traditional ORB algorithm. Figure 6 shows that for the traditional ORB image feature point extraction and matching algorithm, the light field full-focus image obtained in this paper has more feature point matching results and more matching pairs than the light field central aperture image. Especially in the edge part of the image, most of the feature points extracted using the light field central subaperture are in the center part of the image, and the number of the edge parts is smaller, while the feature points extracted using the light field full-focus image basically cover the edge part of the image. For the improved ORB image feature point extraction and matching algorithm, the light field full-focus image feature point matching results are also richer than the image feature point matching results obtained using the traditional ORB algorithm, and the number of matched pairs is also greater. Moreover, the improved ORB image feature point extraction and matching algorithm extracts not only most of the features in the target scene but also covers the edge part of the image to a greater extent, so that the extracted feature points are evenly distributed, and the extracted feature points are not repeated in a large number in a certain part of the image; this approach eliminates the aggregation phenomenon that exists in the traditional ORB algorithm.

**Figure 8:**
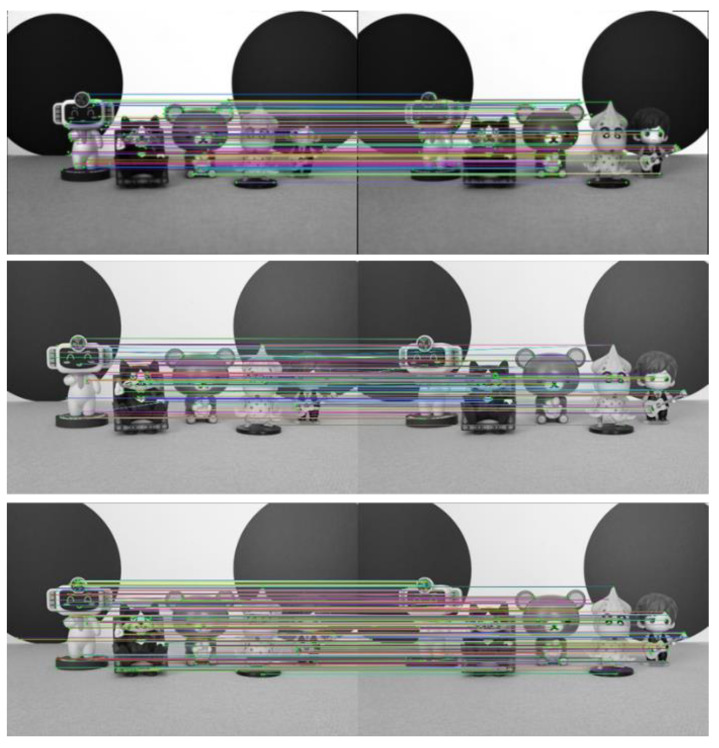
Scene 2 image feature point matching results.

**Figure 9:**
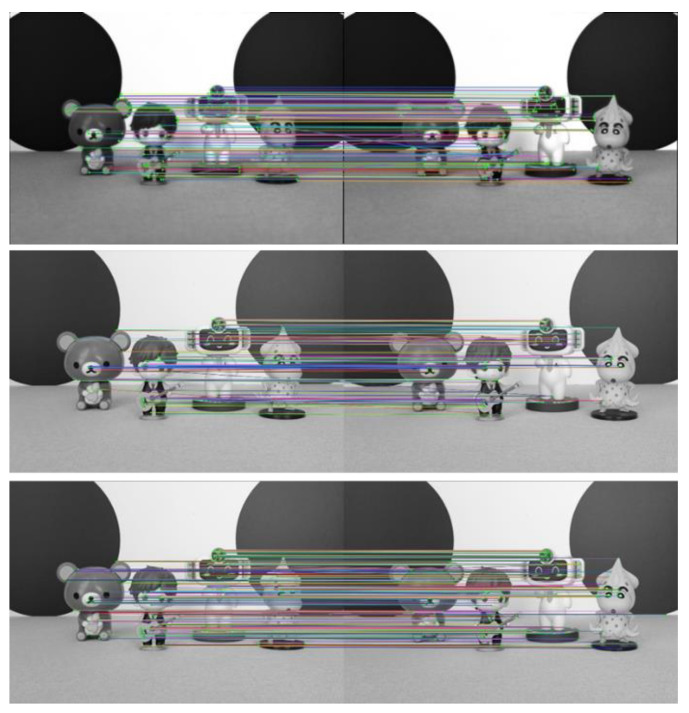
Scene 3 image feature point matching results.

**Figure 10:**
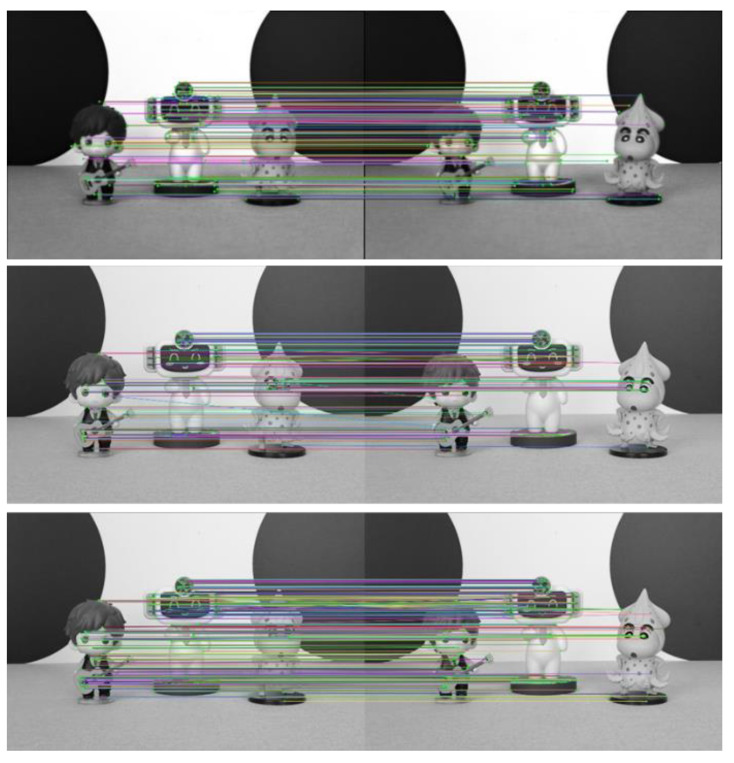
Scene 4 image feature point matching results.

To further verify the effectiveness of the improved ORB algorithm in this paper, experiments are conducted on three other scenes. The experimental results are shown in Figure 8, Figure 9 and Figure 10.

The final feature point matching pairs for the light field central subaperture images and light field full-focus images in the four scenes are shown in Table 2 below.

As seen from Figure 7, Figure 8, Figure 9 and Figure 10 and Table 2, more matching pairs are achieved using the light field full-focus image than using the light field central subaperture image in all four different experimental scenes with the same method. The number of matching pairs achieved using the improved ORB algorithm is also greater than that achieved using the conventional ORB algorithm for the same image. The method in this paper achieves a good matching effect in the edge part of the image, while the other method performs poorly in this aspect, further verifying the effectiveness of the improved ORB algorithm.

The traditional light imaging methods can focus only on a fixed depth of the target scene, and the subject is often blurred at other depths, so most of the feature points that can be extracted from traditional 2D images are gathered at their depth of focus, and some feature points at other depths are often easily missed. This leads to a reduction in the number of final feature matches. The light field full-focus image acquired in this paper is processed on the original image of the light field, which contains rich depth information in the scene, so more feature points can be extracted from the image. This method also increases the number of final feature matches. In addition, the improved ORB algorithm adopts the adaptive threshold method to adjust the restricted range of the feature point extraction and uses the nonmaximal value suppression method to eliminate the shortcoming of the aggregation phenomenon in the extracted feature points in the traditional ORB algorithm. Thus, the extracted feature points are abundant, evenly distributed, and have a large number of matching pairs.

In summary, we improve the ORB image feature extraction and matching algorithm to totally extract the feature points at various depths in the target scene with the help of the rich depth information contained in the light field full-focus image and its robustness in dealing with challenging scenes. The extracted feature points not only have a general scale invariance and rotation invariance but also have unique depth information of the light field image, which makes the number of matching image features more numerous and more evenly distributed.

## 5. Conclusions

Based on the fact that light field imaging technology can obtain a series of images focused at different depths by only one exposure process, in this paper, an in-depth study on the generation of light field full-focus images is conducted using the original light field images. The traditional ORB feature point extraction and matching algorithm is enhanced with the goal of improving the number and accuracy of the feature point extraction for light field full-focus images. The improved ORB algorithm extracts not only most of the features in the light field full-focus image but also covers the edge part of the image to a greater extent so that the extracted feature points are evenly distributed in all parts of the image. Moreover, the extracted feature points are not repeated in a large number in a certain part of the image, eliminating the aggregation phenomenon that exists in the traditional ORB algorithm.

The shortcomings of this paper are as follows.

(1) Due to the limitation of the topic and space, for the light field full-focus images acquired by the method in this paper, only a relatively simple image sharpness evaluation function is used to evaluate their quality, and a more comprehensive image quality evaluation method is not used to evaluate them.
(2) Due to the specificity of the built-in sensor of the light field camera, the method in this paper has not been tested on the existing open dataset.

In future research, we will investigate this in more detail and take more light field images of scenes to establish a dataset to verify the generalizability of the method in this paper.

## Figures and Tables

**Table 1:** Image clarity evaluation Tenengrad value (Retain two decimal places).

	Scene 1	Scene 2	Scene 3	Scene 4
Light field full-focus image	43.70	47.54	40.26	38.40
Light field central subaperture image	38.34	45.13	35.96	35.83

**Table 2:** Number of matching pairs of image feature points.

	Scene 1	Scene 2	Scene 3	Scene 4
Traditional ORB algorithm light field central subaperture image	112	109	109	113
Traditional ORB algorithm light field full-focus image	190	172	125	179
Improved ORB algorithm light field full-focus image	739	578	466	659

## Data Availability

Not applicable.
